# Substituent Effects on the Ultraviolet Absorption Properties of 2,4-Dihydroxy Dibenzophenone

**DOI:** 10.3390/molecules27238169

**Published:** 2022-11-24

**Authors:** Feng Wu, Shengqiong Tan, Zhengjun Fang, Jiyu Deng, Zhengjie He, Chaoyi Huang, Chaktong Au, Bing Yi

**Affiliations:** Hunan Provincial Key Laboratory of Environmental Catalysis & Waste Recycling, College of Materials and Chemical Engineering, Hunan Institute of Engineering, Xiangtan 411104, China

**Keywords:** 2,4-dihydroxy dibenzophenone, ultraviolet absorbent, UV resistance, substituent effects

## Abstract

Substituent effects on the ultraviolet absorption properties of 2,4-dihydroxy dibenzophenone were investigated experimentally. Nine compounds of 2,4-dihydroxy dibenzophenone with different substituents were prepared by a solvent-free reaction of benzoyl chloride. The maximum absorption wavelength (λ_max_) of these samples was measured, and their UV resistance properties in cotton fabric as well as in polyester were determined. The results show that the λ_max_ is dependent on the substituents at the benzylidene ring, and both electron donating substituents and electron withdrawing substituents cause a bathochromic shift. The UV resistance of fabric increases with the increase in compound concentration. The dyeing rate of each compound on polyester was higher than that of cotton. On cotton fabric, the dyeing rate of 2,4-dihydroxybenzophenone was the highest, 77.8%. On polyester, that of 2,4-dihydroxy-4′-ethyl dibenzophenone was the highest, 84.1%. The study provides new insights into the effect of substituents on the properties of 2,4-dihydroxy dibenzophenone that are related to the whitening of cotton and polyester materials.

## 1. Introduction

With unique physical and chemical properties, ultraviolet absorption agents are widely used in cosmetics [1,2,3], fabric [4,5,6], painting [7,8], UV protection and light stabilizer industries, mainly to protect materials and humans from being damaged by ultraviolet light [9,10]. Designing and fabricating effective UV absorbents by energy saving and environmentally benign methods is a research hotspot.

The absorption spectrum of UV absorbent is mainly from 200 to 400 nm [11,12]. The absorption mechanism of benzophenone is that there is hydrogen bonding between the carbonyl group within the molecule and the hydroxyl group nearby. When the hydrogen bond is broken after absorbing ultraviolet radiation energy, the six-member ring containing the hydrogen bond is opened, and the molecule is transformed from enol to ketone structure. When the ultraviolet radiation is converted to light of longer wavelength and heat, there is the regeneration of hydrogen bond [13,14].

Of the UV absorbent materials, benzophenones were first used, and among them 2,4-dihydroxybenzophenone was the simplest. It has been applied to various kinds of polymer, rubber and coating materials to absorb ultraviolet rays. It is also the basic raw material for the synthesis of UV-9, UV-531, UV-B, etc. [15]. Because of the special value of UV-0, it has been widely researched on.

Research showed that the modification of the parent structure of benzophenone or the introduction of specific groups can bring about a variety of application effects to the UV absorbent. For example, Cao et al. [16] acylated benzoic acid with resorcinol, and further sulfonated the intermediate with concentrated sulfuric acid to prepare 2,4-dihydroxy-5-sulfonic acid dibenzophenone. Because the molecule contains hydrophilic sulfonic acid group, the product is water soluble. Moreover, from UV-0 and chlorooctane under the catalytic action of KI, Tong et al. [17] synthesized 2-hydroxy-4-n-octanoxy dibenzophenone (UV-513) with increased alkoxy chain length, and the outcome is significant improvement of thermal stability and better resin compatibility.

To acquire solid evidence of substituent effects on benzophenone, we chose 2,4-dihydroxybenzophenone for such a goal. In this study, nine compounds of 2,4-dihydroxy dibenzophenone with different substituents were synthesized and their ultraviolet absorption performance and UV resistance of cotton fabric and polyester analyzed. Moreover, the dyeing rate of each compound on cotton fabric and polyester were tested, respectively. The outcome of the present study is anticipated that a research basis for the development and design of benzophenone UV absorbents.

## 2. Experimental Section

### 2.1. Sample Preparation

**Scheme 1:**
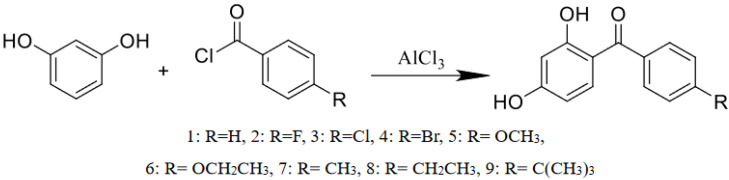
Synthetic route of 2,4-dihydroxy dibenzophenone compounds.

All the chemicals were purchased from Aladdin Industrial Corporation (Shanghai, China), and used as received. Compounds **1**–**9** shown in Scheme 1 were synthesized by a solventless method.

First, 50 mmol of resorcinol was heated at 140 °C and 15 mmol of anhydrous aluminum chloride was added to the molten resorcinol, and the mixture was stirred until complete mixing. Then 10 mmol of *p*-substituted benzoyl chloride was added, and the as-resulted mixture was stirred in its molten state for 10 min for the generation of a solid powder. Upon cooling to room temperature, a cold aqueous solution of hydrochloric acid (50:1) was added with stirring. After the quenching step, the final mixture was subject to extraction with ethyl acetate four times. The combined organic layer was dried over MgSO_4_, and after evaporation and recrystallization from anhydrous ethanol/water mixed solvent, there was the generation of the target compound in high purity. The product was vacuum dried at 60 °C overnight. The ^1^H NMR and ^13^C NMR spectra are provided in Supplementary Materials.

### 2.2. Pretreatment of Fabrics

**Figure 1:**
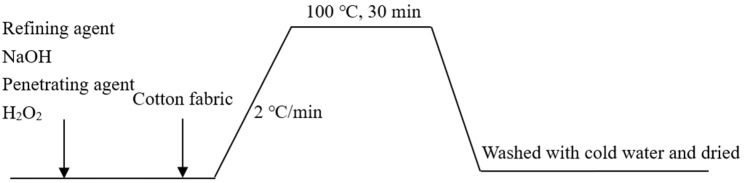
Pretreatment process of cotton fabric.

**Figure 2:**
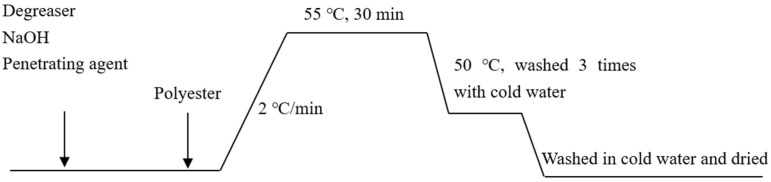
Pretreatment process of polyester.

Cotton fabric and polyester were used as test materials and were cut to specifications. Cotton cloth not only contained slurry and oil stains that needed spinning and weaving but also contained natural impurities such as cotton seed shells, which needed pretreatment such as desizing, scouring and bleaching [18]. Polyester, on the other hand, only needed a pretreatment of simple oil-removing [18]. The processes are as depicted in Figure 1 and Figure 2.

### 2.3. UV Resistance Test

The fabrics for the test were coated with the absorbent by impregnation method. The weight of fabric used was 1.0 g, and the absorbent solutions prepared were 0, 10^−l^, 5.0 × 10^−4^, and 2.5 × 10^−4^ mol of each of the prepared compound in 50 mL of anhydrous ethanol. The fabric was soaked in one of the above solutions at room temperature for 1 h. The fabric was fully soaked before the test. For full coating of the UV absorbent, the fabrics were taken out, and after natural drying, went through the coating procedure for a total of three times (disposable gloves were used). The fabric coated with UV absorbent was cut into the required round shape and tested.

### 2.4. Dyeing Rate Test

First, 0.01 mmol of UV absorbent was accurately weighed and dissolved (with shaking) in 100 mL of anhydrous ethanol. Then, 2.0, 4.0, 6.0, 8.0, and 10.0 mL of the prepared UV absorbent solution was separately transferred to a 50 mL volumetric flask and made up to 50 mL with anhydrous ethanol. Taking anhydrous ethanol as the standard solution, the scanning wavelength range from 200 to 400 nm was obtained.

## 3. Results and Discussion

### 3.1. Effects of Substituents on UV Spectra of 2,4-Dihydroxy Dibenzophenone

**Figure 3:**
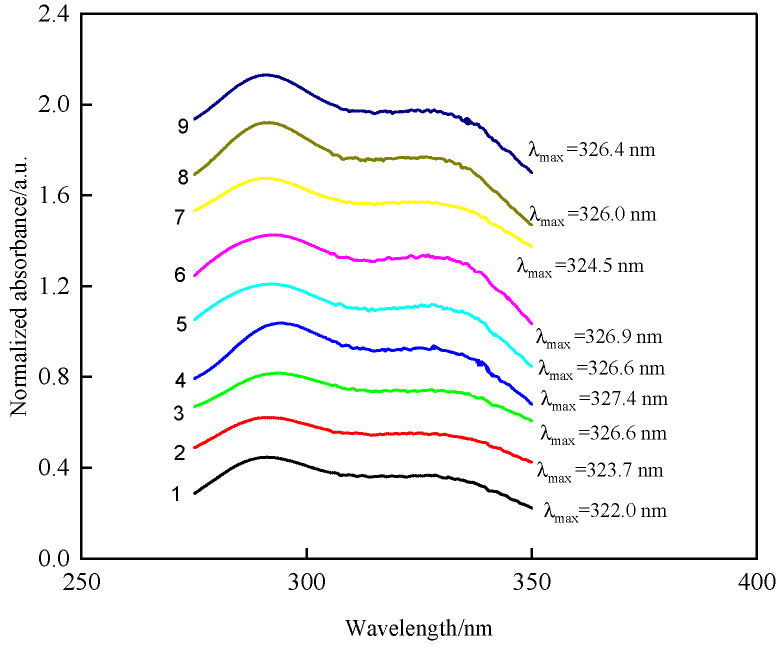
Normalized absorption spectra for the nine compounds in ethanol.

The UV absorption spectra of the nine compounds were collected at room temperature on a UV-1800 UV-vis spectrometer having 2,4-dihydroxy dibenzophenone in a concentration range of 10^−3^ to 10^−5^ mol/L. The ethanol used in the experiments was of spectroscopic grade purchased from Aladdin Industrial Corporation (Shanghai, China) and was used as purchased. The ultraviolet spectra are shown in Figure 3. For comparison purposes the maximum absorbance is normalized for all measurements. 

Using the same solvent and with increasing electron withdrawing or electron donating ability of substituents, the compounds display a bathochromic shift. The observation is in accord with the phenomena of recent studies [19,20,21]. As can be seen, λ_max_ ranges from 322.0 to 327.4 nm across the compounds. Compound **4** has a maximum λ_max_ of 327.4 nm, whereas that of Compound **1** is only 322.0 nm. A comparison of the λ_max_ values across the compounds with different substituents reveals that λ_max_ is not only under the influence of the backbone of the compound, but also sensitive to the substituents. The absorption signals in the range of 322.0–327.4 nm is assigned to the intramolecular charge transfer band of the compounds [22].

### 3.2. Effects of Substituents on UV Resistance of 2,4-Dihydroxydibenzophenone

The results of the test are compiled in Table 1. When the concentration of UV absorbent is 2 × 10^−2^ mol·L^−1^, the UPF (ultraviolet protection factor) of the nine UV absorbents is larger than 40, reaching the national standard. The higher the concentration of UV absorbent, the higher the UPF value of coated cotton fabric, and the lower the T(UVA), T(UVB), T(UVR), the better the UV resistance. 

In the absence of substituents, the effect of absorbers on the UV resistance effect of fabrics was significantly weaker than that of substituents, indicating that the different substituents greatly improved the performance of benzophenone UV absorbers.

The fabric treated with 2,4-dihydroxybenzophenone has the highest UPF value, the lowest T(UVA) and the best UV resistance. The reason may be that the 2,4-dihydroxybenzophenone molecule is the simplest in structure, having good coplanar properties and strong van der Waals force between the UV absorbent and cotton fabric [23]. There is methoxyl and ethoxyl group in compound **5** and **6**, respectively. With the increase in molecular chain length, the adsorption effect of UV absorbent on cotton fabric worsened.

The number of hydroxyl groups affected the UV resistance of cotton fabric to some extent. The influence relationship is roughly proportional, that is, the more the amount of hydroxyl, the better the UV resistance of cotton fabric. In the case of dihydroxyl on benzophenone, the position of the hydroxyl group will also affect the UV resistance effect of polyester fabric. With the increase in the number of hydroxyl group, the effect of absorbent on the UV resistance effect of polyester fabric did not significantly improve, and even weakened.

Because polyester fabric is rich in benzene ring, the fabric by itself has high UV absorption capacity, so the UV resistance of the coated polyester is significantly higher than that of coated cotton. The UPF of polyester treated with the three different concentrations of UV absorbent is higher than the national standard 40. The higher the concentration of UV absorbent, the higher the UPF value, and the lower the T(UVA), T(UVB), T(UVR), confirming better UV resistance of polyester. The effect of 2,4-dihydroxybenzophenone on the UV resistance of polyester fabric is slightly lower than that endowed with substituents. It can be seen that the presence of substituents can improve the UV absorption performance of 2,4-dihydroxybenzophenone to some extent. The presence of methoxide does not significantly improve the UV resistance of polyester.

### 3.3. Dyeing Rate of 2,4-Dihydroxybenzophenone UV Absorbent Coated on Fabrics

Taking absorbance as ordinate and concentration as abscissa, the standard curve of absorbance versus concentration was provided in Supplementary Materials. The data of the test are compiled in Table 2.

From the standard curves, it is obvious that the absorbance of the nine 2,4-dihydroxy dibenzophenone UV absorbents increases with the increase in solution concentration. The dyeing rate of the compound on the fabric can be calculated by measuring the absorbance of the solution before and after soaking the fabric. The calculation formulas are as follows:

Concentration: c = x × 2 × 10^−6^ × 10,000

Dyeing rate: η = c × 50 × 10^−3^/0.001 mol × 100%

The correlation coefficient *R*^2^ of all standard curves is larger than 0.9990. The staining rates (η) of the nine UV absorbents are all greater than 65% (except for **5**, **6** and **9**), but there are still high requirements for waste-liquid treatment. The dyeing rate of 2,4-dihydroxy dibenzophenone on cotton fabric was 77.8%, which was the highest among the nine absorbents. The reason may be that the molecular structure is the simplest, and the coplanar property is suitable for easy and direct adsorption on the surface of cotton fabric, resulting in an increase in dyeing rate. Group **5**, **6** and **9** contain methoxyl, ethoxyl and tert-butyl, respectively. With the increase in molecular chain length, there was weakening of absorptivity on cotton, and hence the decrease in dyeing rate.

The dyeing rate of the nine UV absorbents on polyester is higher than that of cotton. The dyeing rates of compounds **7**, **8** and **9** are higher than 80%. It is because the molecule contains methyl, ethyl and tert-butyl group, respectively, leading to an increase in molecular chain length to a certain extent. The phenomenon is like that of dye dispersion, and according to the principle of “similar miscibility”, it can be viewed as “dissolved” in the amorphous region of the fabric [24]. It is considered that there was a reduction in terms of the migration of dye compounds, leading to better absorption effect on polyester. Nonetheless, the molecular weight of the compound should not be too large, otherwise there will be an increase in diffusion activation energy and a decrease in permeability of the molecules in the amorphous region of the fabrics.

## 4. Conclusions

The benzoyl chloride method is the most common for the synthesis of dibenzophenone with different substituents. The best solvent-free reaction conditions for 2,4-dihydroxybenzophenone formation (yield = 69.8%) are (i) resorcinol:benzoyl chloride:aluminum chloride molar ratio of 5:1:1.2, (ii) at 140 °C and (iii) with stirring for 8 min. All the nine sample compounds had absorbance in the range of 200 to 400 nm, among them, 2,4-dihydroxy-4’-bromodibenzophenone has a maximum λ_max_ of 327.4 nm, whereas that of 2,4-dihydroxybenzophenone is only 322.0 nm. A comparison of the λ_max_ values across the compounds with different substituents reveals that λ_max_ is not only under the influence of the backbone of compound but also sensitive to the substituents. The UPF values of cotton fabric and polyester increase with the increase in compound concentration. For cotton fabric, when the concentration of UV absorbent is 2 × 10^−2^ mol·L^−1^, 2,4-dihydroxybenzophenone has the best UV resistance of 143.2, but its UV resistance for polyester is slightly lower than that endowed with the substituted groups. The presence of the methoxyl group does not significantly improve the UV resistance of polyester. It is envisaged that through the selection of substituent, the UV resistance properties of 2,4-dihydroxybenzophenone can be tuned for specific applications.

## Figures and Tables

**Table 1:** UV resistance of fabric.

Concentration/mol·L^−1^	Compound No.	Cotton Fabric				Polyester			
		UPF	T(UVA)%	T(UVB)%	T(UVR)%	UPF	T(UVA)%	T(UVB)%	T(UVR)%
0	blank	30.7	4.0	2.9	3.6	89.7	3.8	0.4	2.8
2 × 10^−2^	**1**	61.9	2.4	1.3	2.0	136.7	2.3	0.4	1.8
	**2**	53.6	2.6	1.5	2.3	143.2	2.4	0.4	1.8
	**3**	52.5	2.5	1.5	2.2	142.9	2.1	0.4	1.6
	**4**	52.1	2.7	1.5	2.4	112.5	2.6	0.5	2.0
	**5**	48.0	2.8	1.7	2.4	132.4	2.7	0.4	2.0
	**6**	45.4	3.0	1.7	2.7	141.1	2.3	0.4	1.7
	**7**	47.1	3.0	1.7	2.6	137.9	2.4	0.4	1.8
	**8**	46.7	3.1	1.7	2.7	138.4	1.8	0.5	1.4
	**9**	42.3	3.2	1.9	2.8	138.5	1.9	0.5	1.5
1 × 10^−2^	**1**	51.1	2.8	1.5	2.4	115.4	2.6	0.5	2.0
	**2**	48.9	2.9	1.6	2.5	127.0	2.5	0.4	1.9
	**3**	41.1	3.3	1.9	2.9	125.0	2.7	0.4	2.0
	**4**	37.4	3.4	2.1	3.0	93.8	3.5	0.6	2.6
	**5**	38.7	3.3	2.1	2.9	104.8	3.1	0.5	2.3
	**6**	39.2	3.4	2.0	3.0	125.3	2.8	0.4	2.1
	**7**	42.4	3.2	1.9	2. 8	118.4	2.4	0.5	1.8
	**8**	42.8	3.1	1.9	2.8	122.7	2.3	0.5	1.8
	**9**	40.1	3.5	2.0	3.1	126.7	2.2	0.5	1.7
5 × 10^−3^	**1**	43.2	3.2	1.8	2.8	103.2	2.9	0.6	2.2
	**2**	44.5	3.1	1.8	2.7	96.0	3.3	0.6	2.5
	**3**	36.4	3.5	2.2	3.1	93.3	3.7	0.6	2.8
	**4**	33.2	3.4	2.4	3.1	91.2	3.1	0.6	2.4
	**5**	35.3	3.6	2.3	3.2	98.2	3.4	0.6	2.6
	**6**	37.8	3.3	2.1	3.0	101.0	3.3	0.6	2.5
	**7**	40.8	3.3	1.9	2.9	91.8	3.9	0.7	2.3
	**8**	40.5	3.6	1.9	3.1	93.5	3.2	0.6	2.5
	**9**	36.0	3.2	2.2	2.9	96.0	3.2	0.6	2.4

**Table 2:** Residual absorbance and dyeing rate of the nine UV absorbents.

No.	Equation	Cotton Fabric		Polyester	
		Absorbance	η%	Absorbance	η%
**1**	y = 0.0252x + 0.0475, *R*^2^ = 0.9998	0.053	77.8	0.052	80.8
**2**	y = 0.0144x + 0.041, *R*^2^ = 0.9997	0.046	68.5	0.044	81.5
**3**	y = 0.0171x + 0.0463, *R*^2^ = 0.9996	0.052	67.8	0.050	77.9
**4**	y = 0.0246x + 0.059, *R*^2^ = 0.9999	0.068	65.3	0.066	75.3
**5**	y = 0.0239x + 0.0711, *R*^2^ = 0.9999	0.080	63.2	0.078	67.4
**6**	y = 0.0184x + 0.0698, *R*^2^ = 0.9998	0.077	60.9	0.075	71.0
**7**	y = 0.0213x + 0.0468, *R*^2^ = 0.9999	0.053	73.2	0.051	83.6
**8**	y = 0.0192x + 0.0561, *R*^2^ = 0.9999	0.062	68.9	0.059	84.1
**9**	y = 0.0145x + 0.0482, *R*^2^ = 0.9999	0.054	61.0	0.051	83.8

## Data Availability

Not applicable.

## Supplementary Materials

The following supporting information can be downloaded at: https://www.mdpi.com/article/10.3390/molecules27238169/s1, Data for ^1^H NMR and ^13^C NMR of compounds **1**–**9**. Spectrum of ^1^H NMR and ^13^C NMR of compounds **1**–**9**. The standard curve of absorbance versus concentration of compounds **1**–**9**.
