# Supplementary material for: Substituent Effects on the Ultraviolet Absorption Properties of 2,4-Dihydroxy Dibenzophenone
Source: Molecules. 2022 Nov 24;27(23):8169. doi: 10.3390/molecules27238169 (PMC9737593; doi:10.3390/molecules27238169)
Supplement: Supplementary file 1 [file molecules-27-08169-s001.zip › molecules-2026688-supplementary.pdf]

SUPPORTING INFORMATION

for

Substituent Effects on the Ultraviolet Absorption Properties of 2,4-Dihydroxy Dibenzophenone

Feng Wu, Shengqiong Tan, Zhengjun Fang *, Jiyu Deng, Zhengjie He, Chaoyi Huang, Chaktong Au and Bing Yi *

(Hunan Provincial Key Laboratory of Environmental Catalysis & Waste Recycling, College of Materials and Chemical Engineering, Hunan Institute of Engineering, Xiangtan 411104, China)

Contents

1. Data for ^{1}H NMR and ^{13}C NMR of compounds 1-9……………………….........……..............2
2. Spectrum of ^{1}H NMR and ^{13}C NMR of compounds 1-9……………………….........………...4
3. The standard curve of absorbance versus concentration of compounds 1-9…………..…....13

1、 **Data for ^{1}H NMR and ^{13}C NMR of compounds 1-9.**

（1）$C_{13}H_{10}O_3$

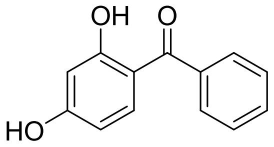

Yield: 69.8%. ^{1}H NMR (500 MHz, $CDCl_3$) δ 12.61 (s, 1H), 7.64 – 7.62 (m, 2H), 7.57 (t, J = 7.5 Hz, 1H), 7.49 (t, J = 7.5 Hz, 3H), 6.47 – 6.47 (m, 1H), 6.35 (dd, J_1 = 10.0, J_2 = 5.0 Hz, 1H), 5.85 (s, 1H).

^{13}C NMR (125 MHz, $CDCl_3$) δ 200.22, 166.05, 138.14, 136.07, 131.52, 130.13, 128.81, 128.31, 113.32, 107.74, 103.60.

（2）$C_{13}H_9FO_3$

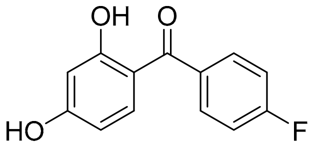

Yield: 42%. ^{1}H NMR (400 MHz, DMSO) δ 11.97 (s, 1H), 10.69 (s, 1H), 7.71 (q, J = 4.0, 2H), 7.37 – 7.33 (m, 3H), 6.40 – 6.37 (m, 2H).

^{13}C NMR (100 MHz, DMSO) δ 197.05, 164.69, 163.98, 163.96 (d, J = 248.0 Hz), 135.00, 134.59 (d, J = 2.0 Hz), 131.49 (d, J = 9.0 Hz), 115.38 (d, J = 22.0 Hz), 112.79, 108.19, 102.75.

（3）$C_{13}H_9ClO_3$

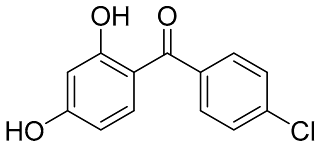

Yield: 51%. ^{1}H NMR (400 MHz, DMSO) δ 11.93 (s, 1H), 10.72 (s, 1H), 7.65 – 7.63 (m, 2H), 7.60 – 7.58 (m, 2H), 7.35 (d, J = 8.0 Hz, 1H), 6.40 – 6.37 (m, 2H).

^{13}C NMR (100 MHz, DMSO) δ 197.11, 164.84, 164.00, 136.79, 136.39, 135.00, 130.54, 128.44, 112.73, 108.27, 102.75.

（4）$C_{13}H_9BrO_3$

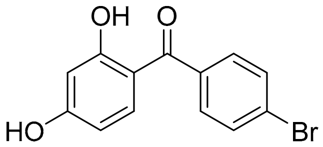

Yield: 50%. ^{1}H NMR (400 MHz, DMSO) δ 11.92 (s, 1H), 10.71 (s, 1H), 7.74 – 7.72 (m, 2H), 7.57 –

7.55 (m, 2H), 7.35 (d, *J* = 8.0 Hz, 1H), 6.40 – 6.36 (m, 2H).

^{13}C NMR (100 MHz, DMSO) δ 197.23, 164.84, 163.99, 137.14, 135.01, 131.37, 130.67, 125.32, 112.70, 108.27, 102.74.

（5）$C_{14}H_{12}O_4$

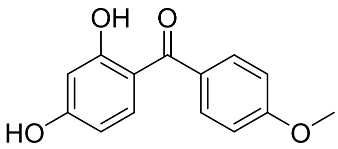

Yield: 55%. ^{1}H NMR (500 MHz, $CDCl_3$) δ 12.61 (s, 1H), 7.66 (d, *J* = 10.0 Hz, 2H), 7.54 (d, *J* = 10.0 Hz, 1H), 6.99 (d, *J* = 5.0 Hz, 2H), 6.46 – 6.46 (m, 1H), 6.36 (dd, J_1 = 10.0, J_2 = 5.0 Hz, 1H), 6.03 (s, 1H), 3.89 (s, 3H).

^{13}C NMR (125 MHz, $CDCl_3$) δ 198.85, 165.86, 162.55, 162.38, 135.73, 131.39, 130.65, 113.64, 108.89, 107.30, 103.67, 55.49.

（6）$C_{15}H_{14}O_4$

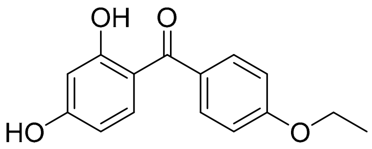

Yield: 48%. ^{1}H NMR (400 MHz, $CDCl_3$) δ 12.63 (s, 1H), 7.64 (d, *J* = 8.0 Hz, 2H), 7.53 (d, *J* = 8.0 Hz, 1H), 6.96 (d, *J* = 8.0 Hz, 2H), 6.46 (d, *J* = 4.0 Hz, 1H), 6.36 (dd, J_1 = 12.0, J_2 = 4.0 Hz, 1H), 4.12 (q, *J* = 6.6 Hz, 2H), 1.45 (t, *J* = 6.0 Hz, 3H).

^{13}C NMR (100 MHz, $CDCl_3$) δ 199.06, 165.78, 162.74, 162.02, 135.84, 131.41, 130.35, 114.13, 113.50, 107.52, 103.65, 63.79, 14.66.

（7）$C_{14}H_{12}O_3$

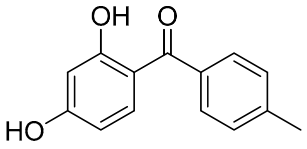

Yield: 41%. ^{1}H NMR (400 MHz, DMSO) δ 12.23 (s, 1H), 10.68 (s, 1H), 7.53 (d, *J* = 8.0 Hz, 2H), 7.39 (d, *J* = 8.0 Hz, 1H), 7.34 – 7.32 (m, 2H), 6.39 – 6.37 (m, 2H), 2.39 (s, 3H).

^{13}C NMR (100 MHz, DMSO) δ 198.45, 164.65, 164.39, 141.79, 135.26, 135.17, 128.87, 128.85, 112.59, 108.06, 102.73, 21.04.

（8）$C_{15}H_{14}O_3$

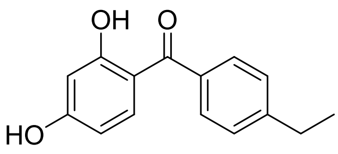

Yield: 40%. ^{1}H NMR (400 MHz, DMSO) δ 12.25 (s, 1H), 10.68 (s, 1H), 7.55 (d, J = 8.0 Hz, 2H), 7.40 – 7.35 (m, 3H), 6.40 – 6.37 (m, 2H), 2.69 (q, J = 6.6 Hz, 2H), 1.21 (t, J = 8.0 Hz, 3H).

^{13}C NMR (100 MHz, DMSO) δ 198.48, 164.69, 164.45, 147.88, 135.51, 135.21, 128.95, 127.71, 112.56, 108.08, 102.74, 28.11, 15.21.

（9）$C_{17}H_{18}O_3$

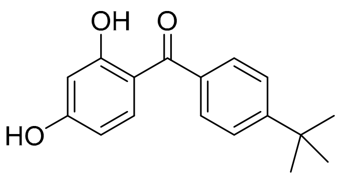

Yield: 36%. ^{1}H NMR (400 MHz, $CDCl_3$) δ 12.72 (s, 1H), 7.58 (d, J = 8.0 Hz, 2H), 7.51 (t, J = 10.0 Hz, 3H), 6.46 (d, J = 4.0 Hz, 1H), 6.37 – 6.34 (m, 1H), 1.36 (s, 9H).

^{13}C NMR (100 MHz, $CDCl_3$) δ 200.37, 165.84, 163.60, 155.33, 136.21, 135.13, 128.88, 125.27, 113.17, 107.95, 103.55, 34.97, 31.07.

2. Spectrum of ^{1}H NMR and ^{13}C NMR of compounds 1-9.

2.1 The ^{1}H NMR spectrum of 2,4-dihydroxybenzophenone

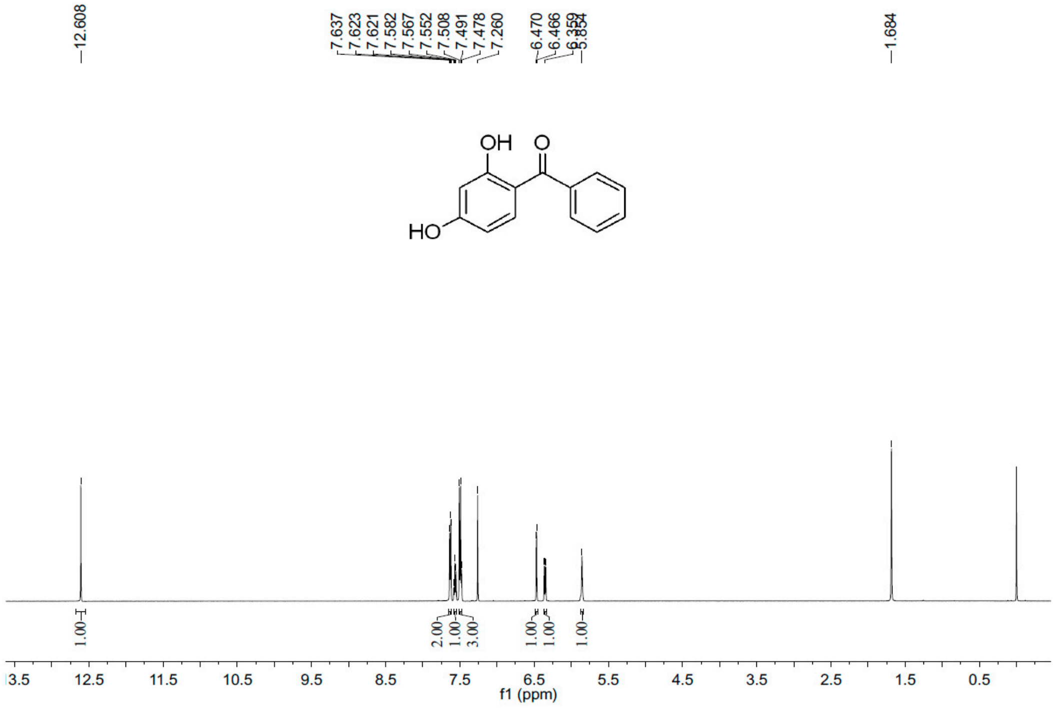

2.2 The ^{13}C NMR spectrum of 2,4-dihydroxybenzophenone

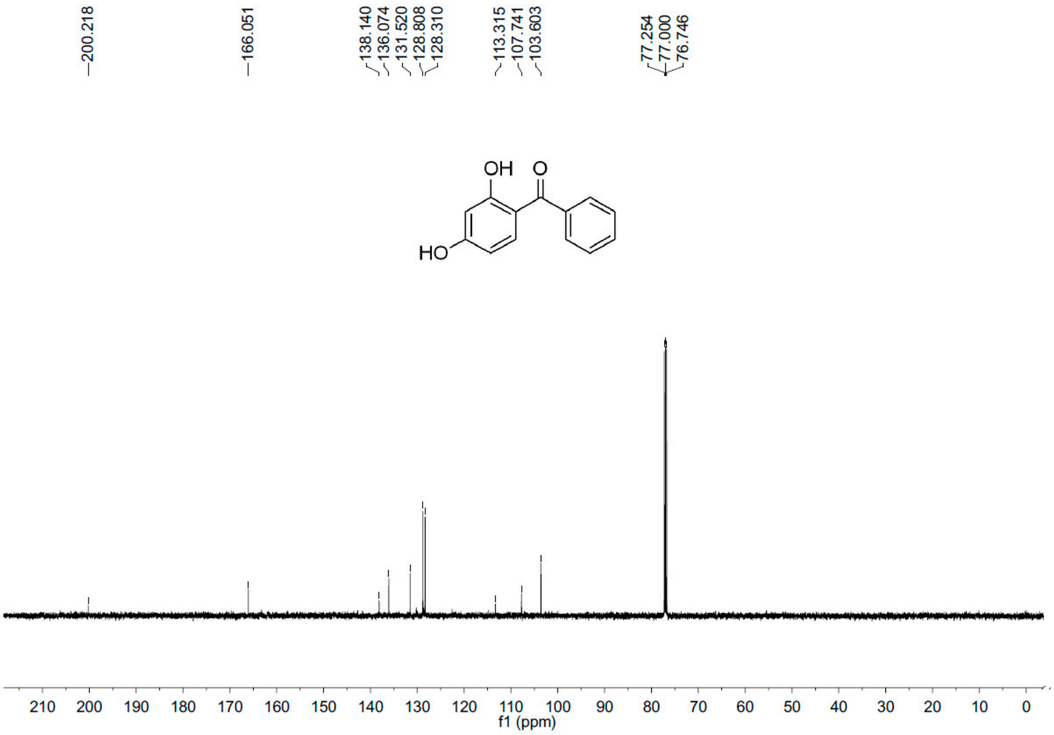

2.3 The ^{1}H NMR spectrum of 2,4-dihydroxy-4'-fluorophenone

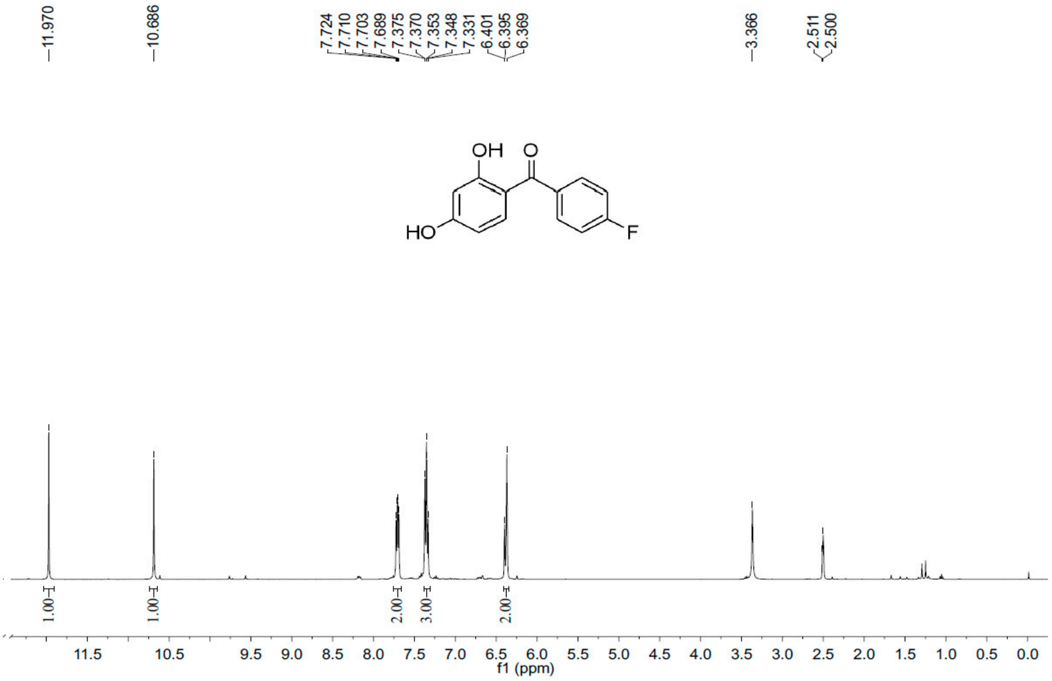

2.4 The ^{13}C NMR spectrum of 2,4-dihydroxy-4'-fluorophenone

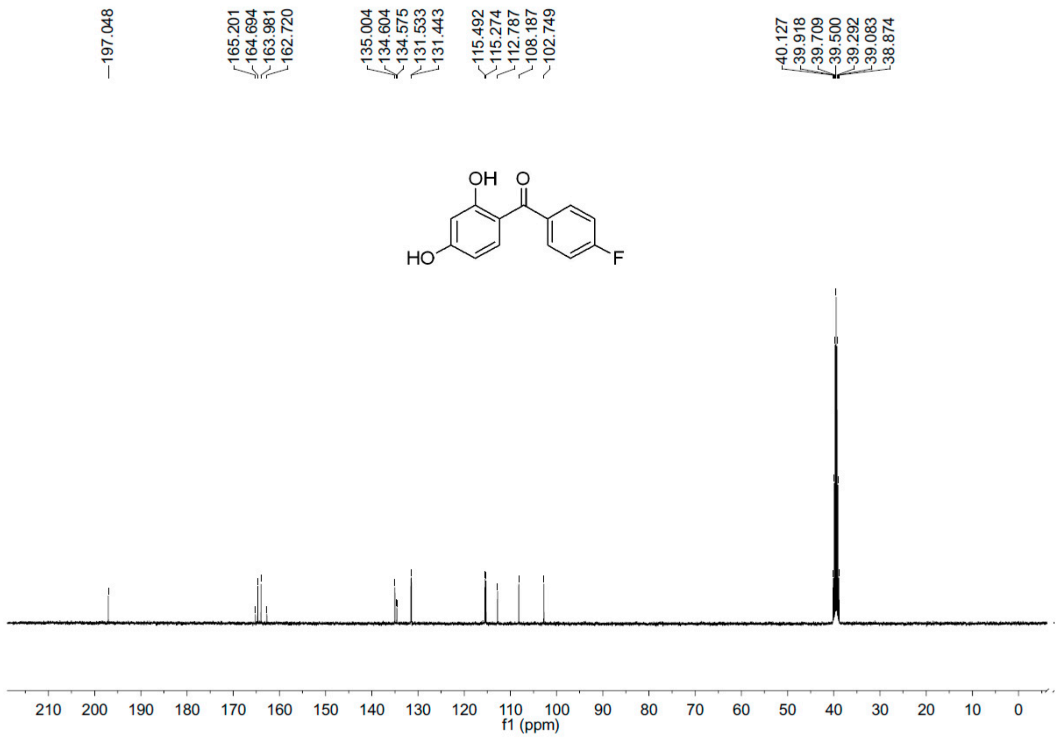

2.5 The ^{1}H NMR spectrum of 2,4-dihydroxy-4'- chlordibenzophenone

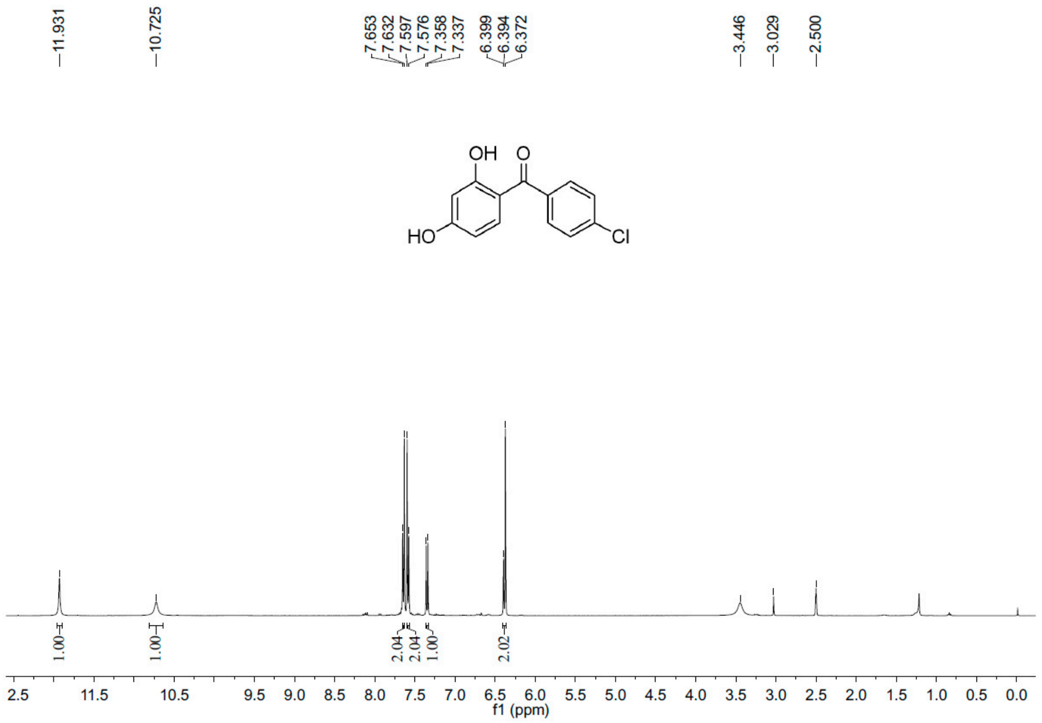

2.6 The ^{13}C NMR spectrum of 2,4-dihydroxy-4'- chlordibenzophenone

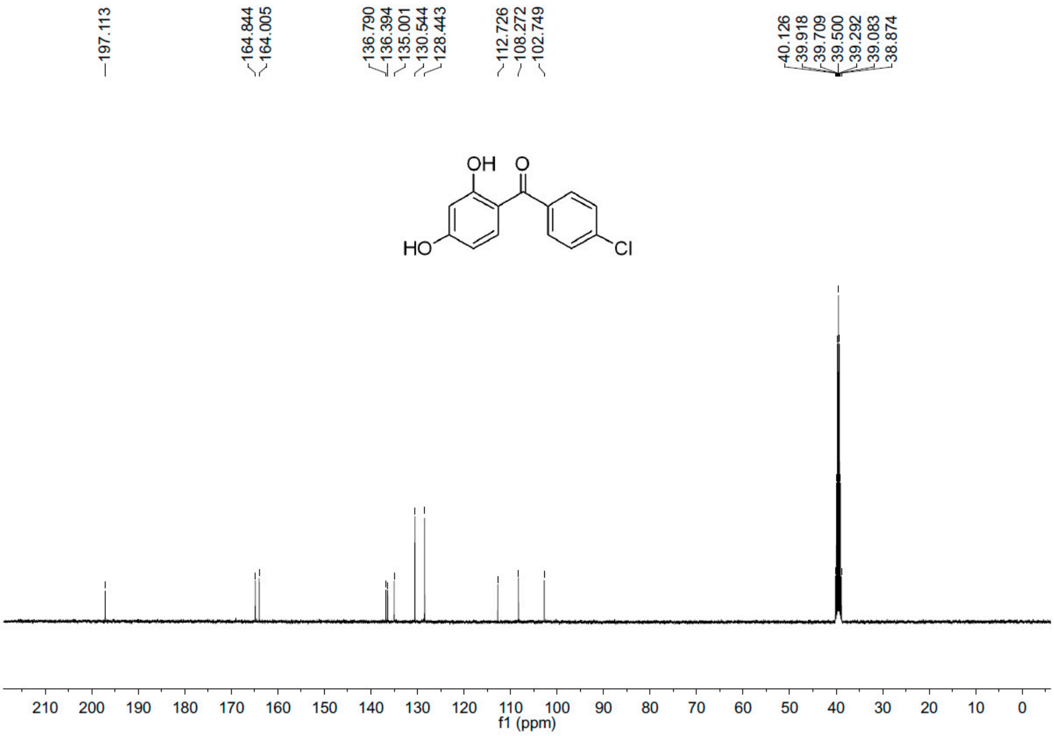

2.7 The ^{1}H NMR spectrum of 2,4-dihydroxy-4'- bromodibenzophenone

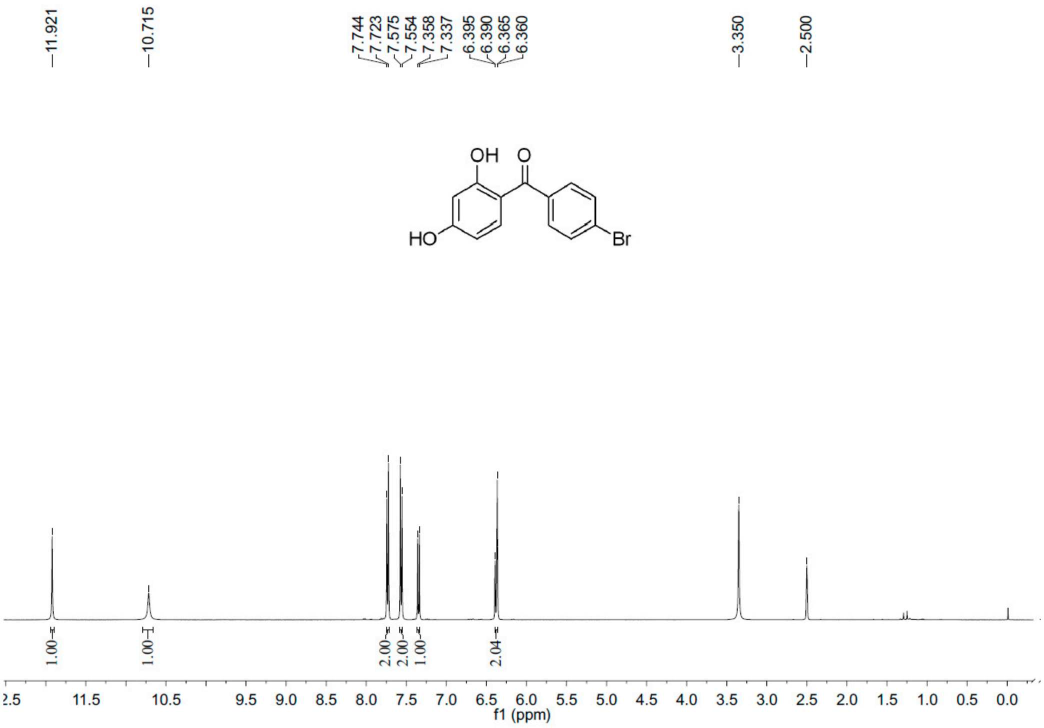

2.8 The ^{13}C NMR spectrum of 2,4-dihydroxy-4'- bromodibenzophenone

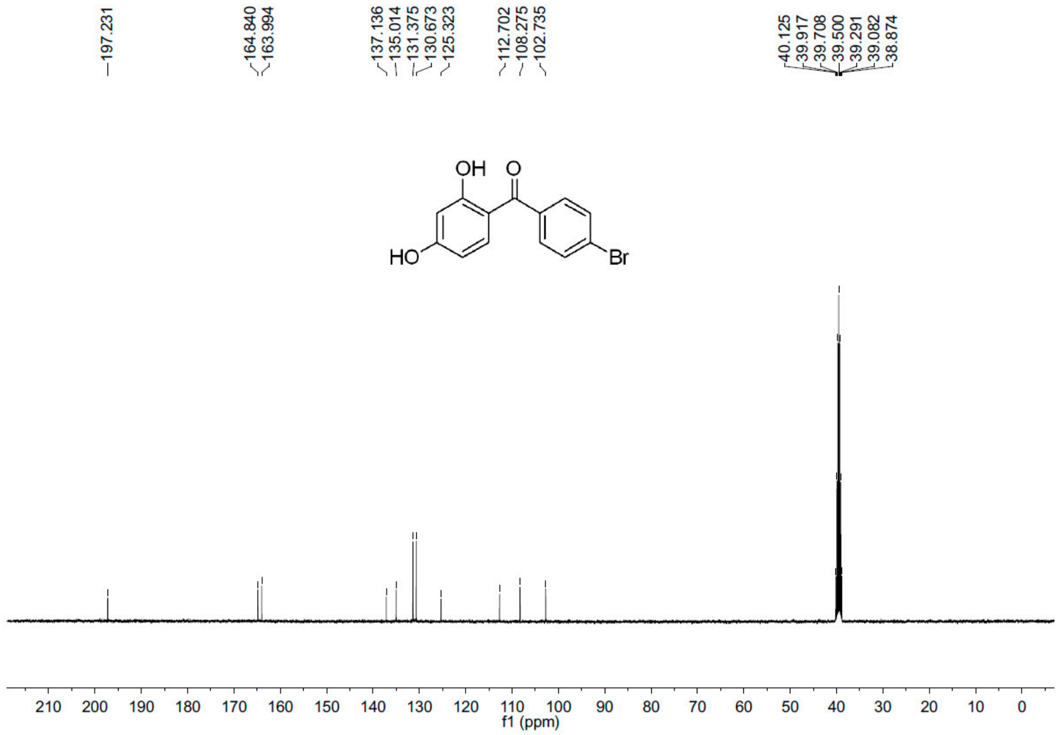

2.9 The ^{1}H NMR spectrum of 2,4-dihydroxy-4'- methoxydibenzophenone

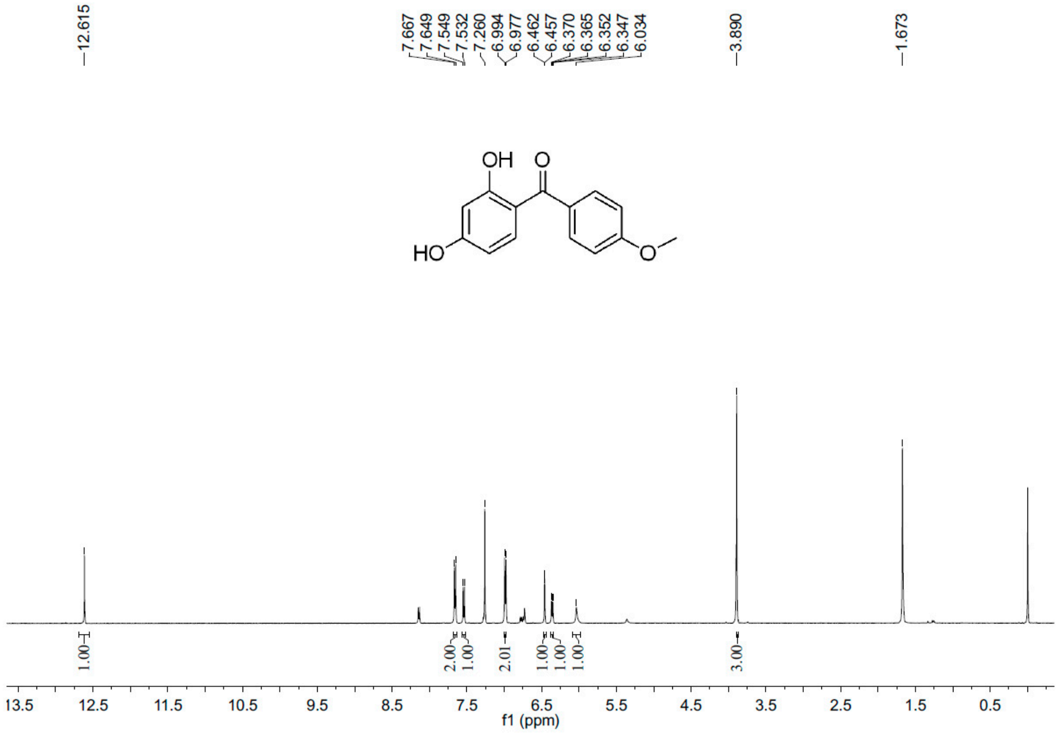

2.10 The ^{13}C NMR spectrum of 2,4-dihydroxy-4'- methoxydibenzophenone

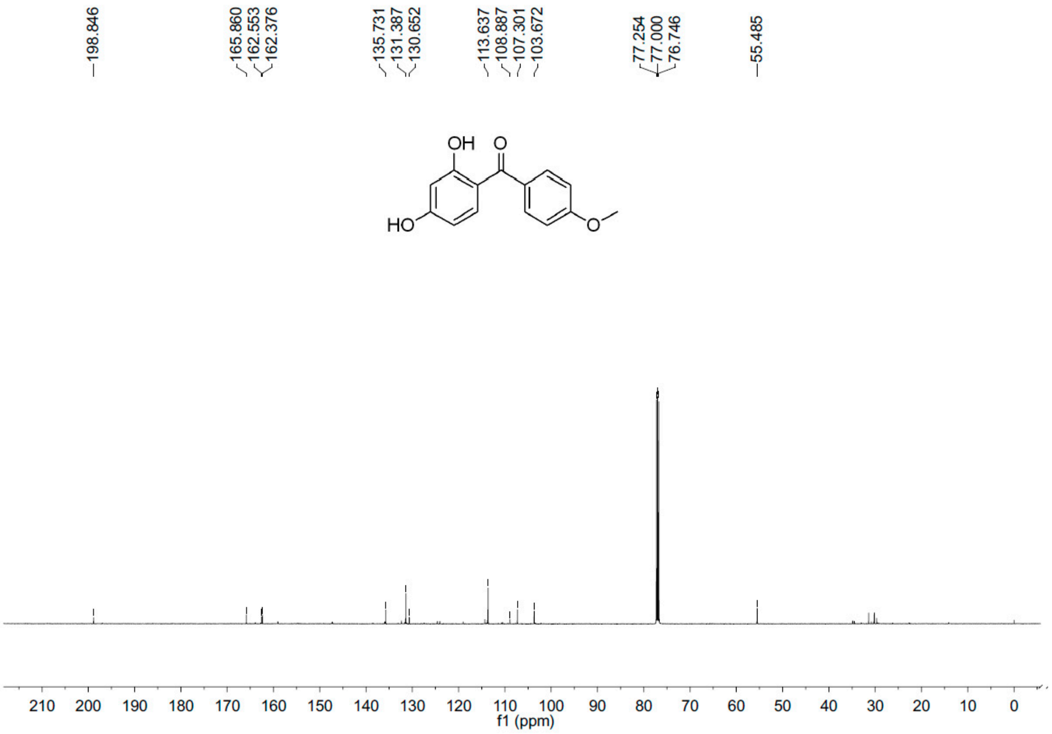

2.11 The ^{1}H NMR spectrum of 2,4-dihydroxy-4'- ethoxydibenzophenone

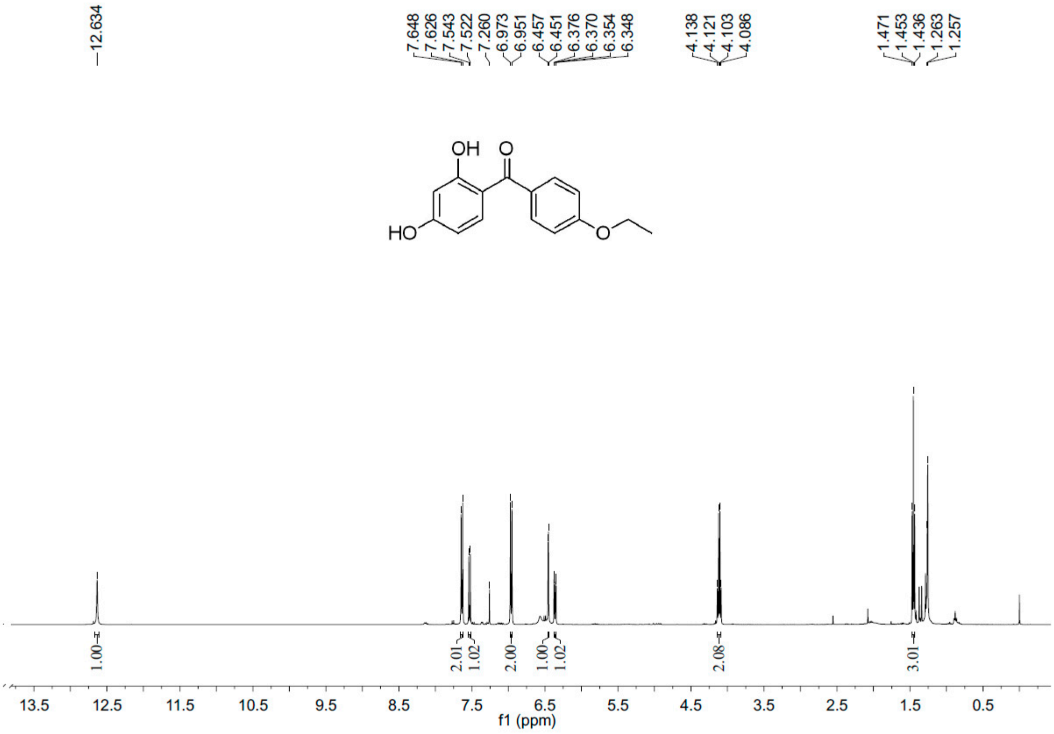

2.12 The ^{13}C NMR spectrum of 2,4-dihydroxy-4'- ethoxydibenzophenone

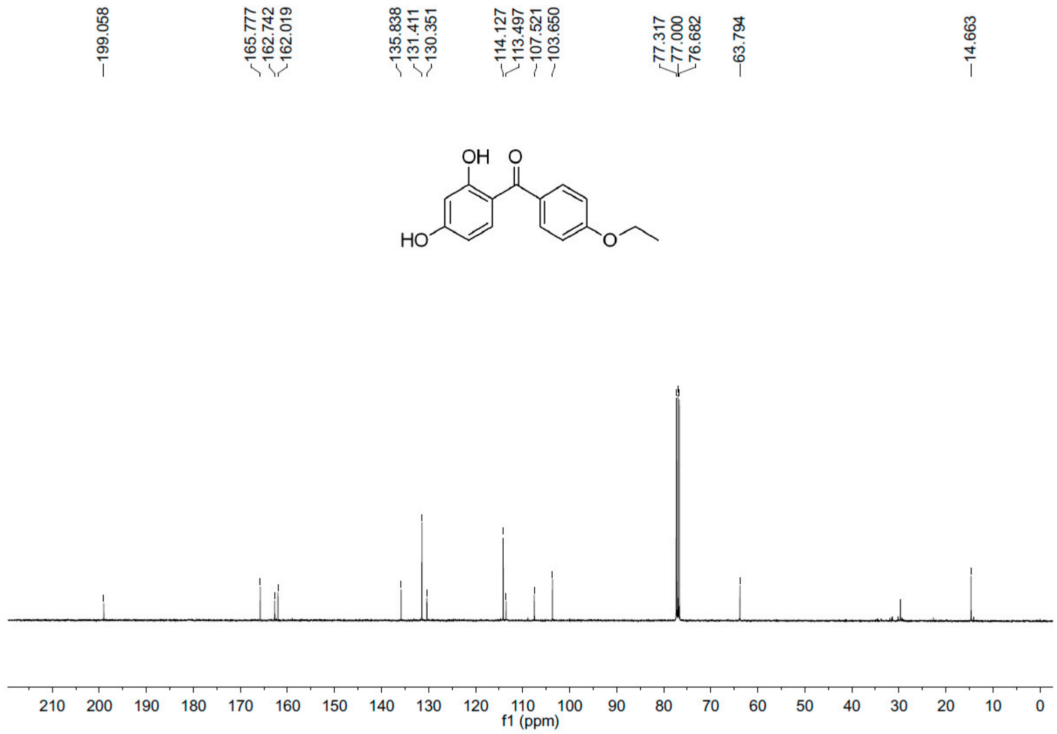

2.13 The ^{1}H NMR spectrum of 2,4-dihydroxy-4' -methyldibenzophenone

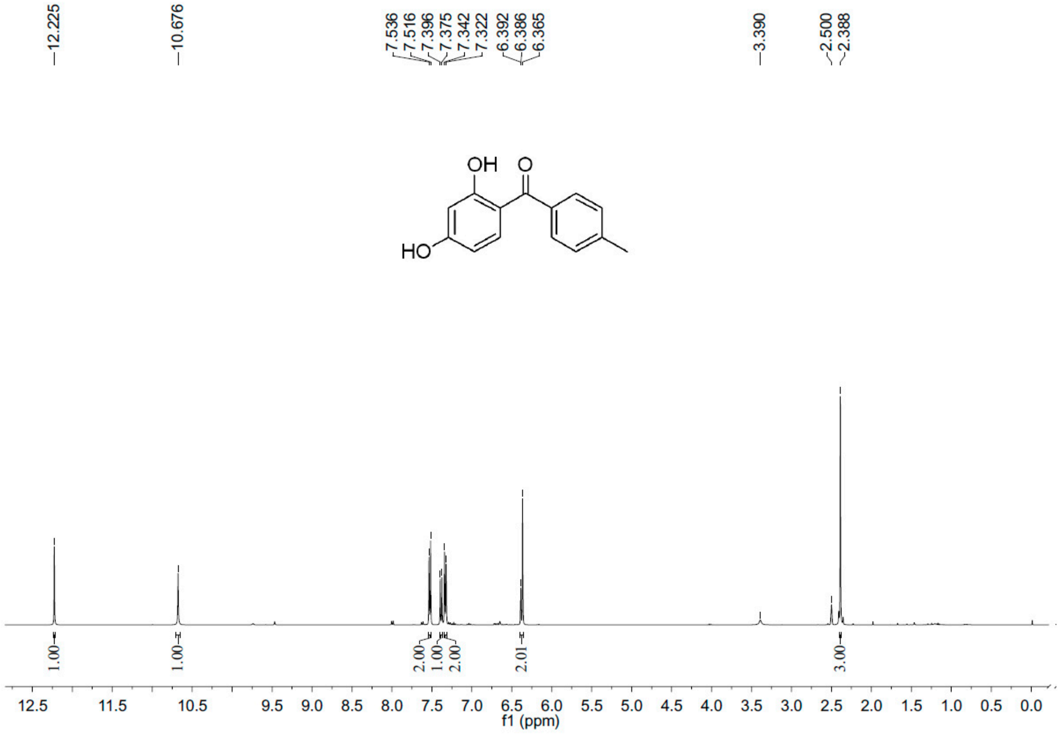

2.14 The ^{13}C NMR spectrum of 2,4-dihydroxy-4' -methyldibenzophenone

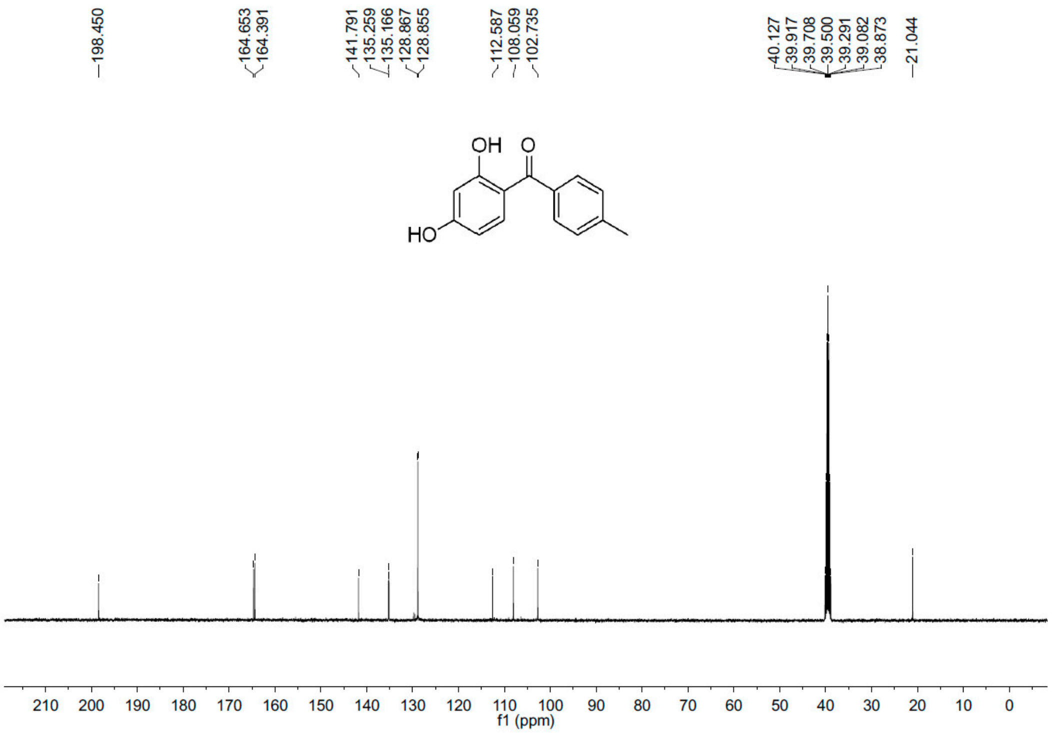

2.15 The ^{1}H NMR spectrum of 2,4-dihydroxy-4' -ethyldibenzophenone

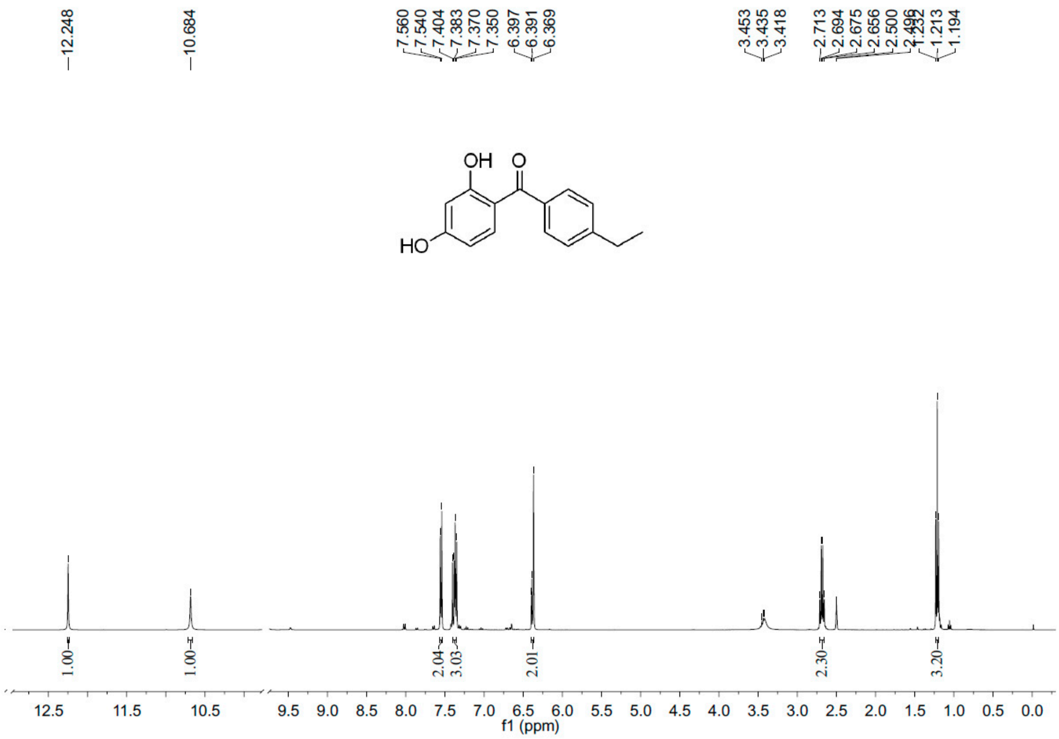

2.16 The ^{13}C NMR spectrum of 2,4-dihydroxy-4' -ethyldibenzophenone

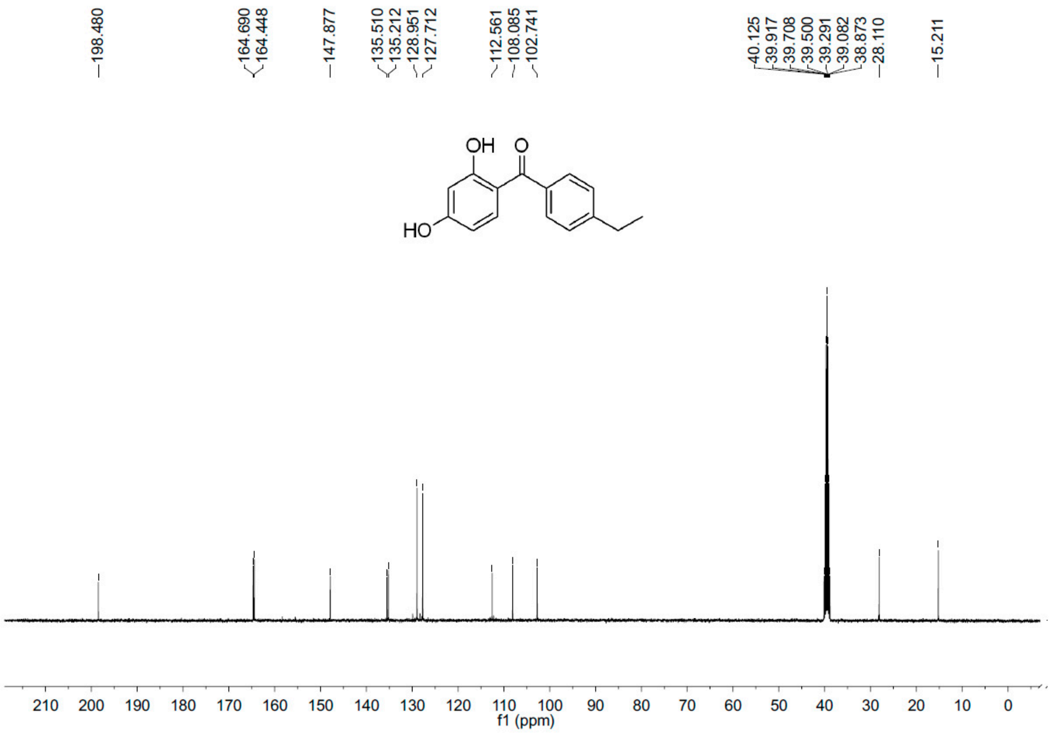

2.17 The ^{1}H NMR spectrum of 2,4-dihydroxy-4'-tert-butyldibenzophenone

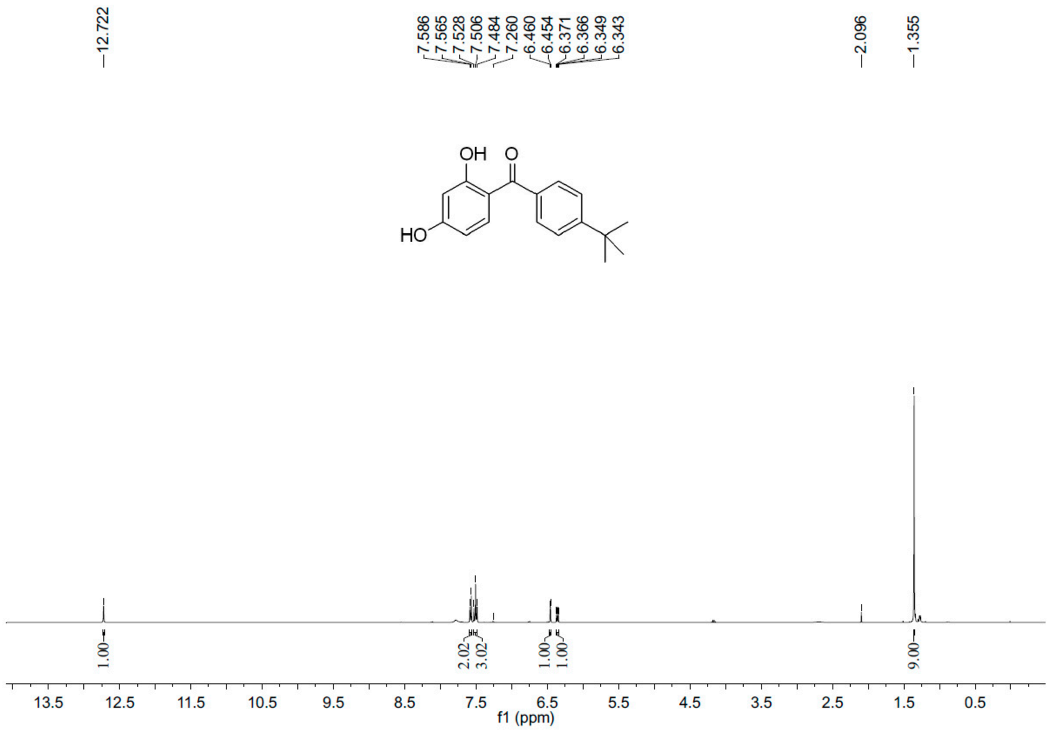

2.18 The ^{13}C NMR spectrum of 2,4-dihydroxy-4'-tert-butyldibenzophenone

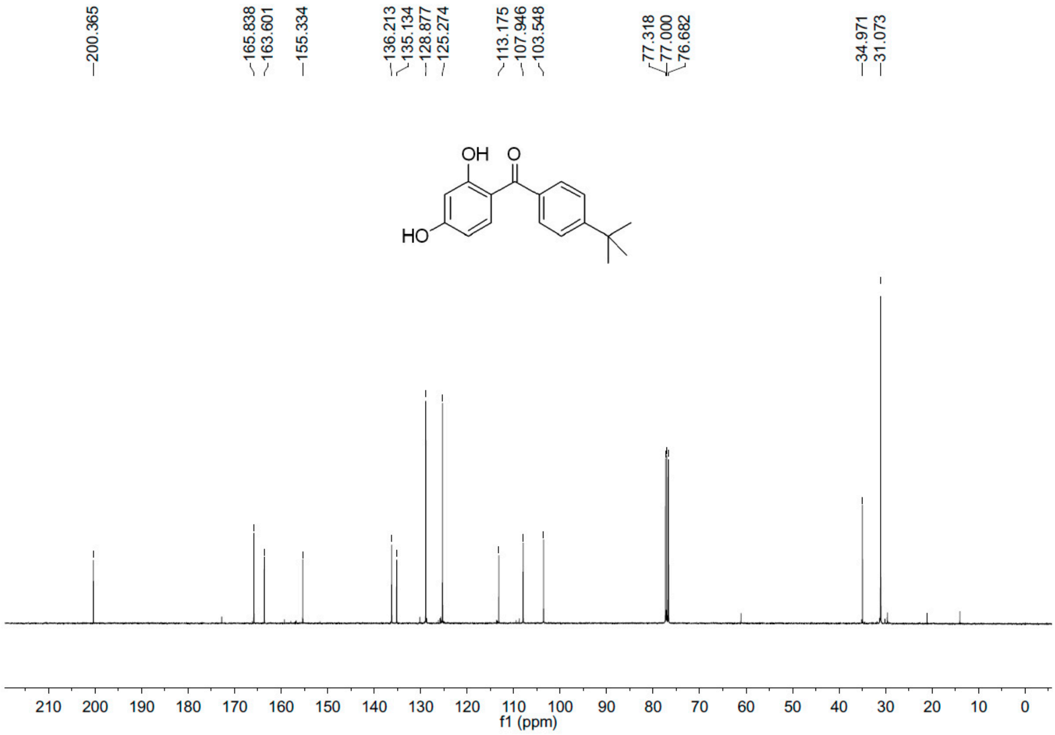

3. The standard curve of absorbance versus concentration of compounds 1-9

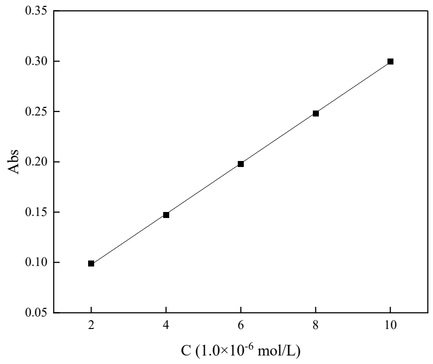

Figure S1. Standard curve of 2,4-dihydroxybenzophenone

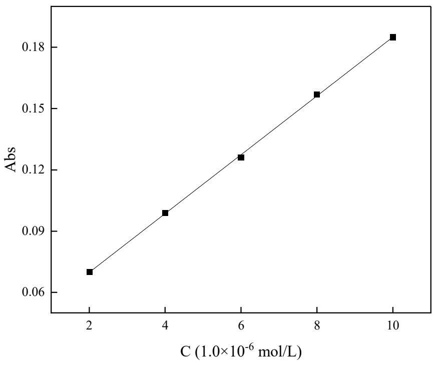

Figure S2. Standard curve of 2,4-dihydroxy-4'-fluorophenone

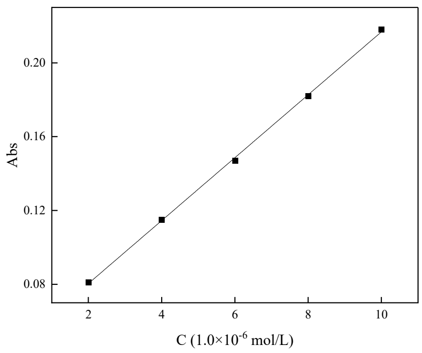

Figure S3. Standard curve of 2,4-dihydroxy-4'-chlordibenzophenone

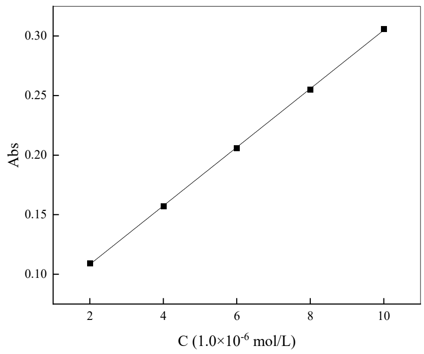

Figure S4. Standard curve of 2,4-dihydroxy-4'-bromodibenzophenone

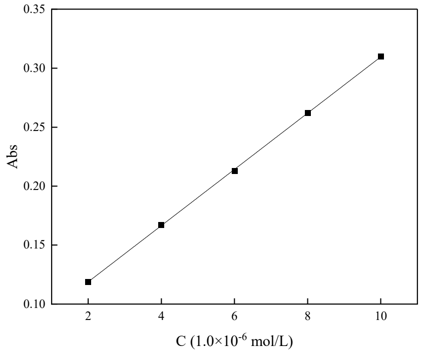

Figure S5. Standard curve of 2,4-dihydroxy-4'-methoxydibenzophenone

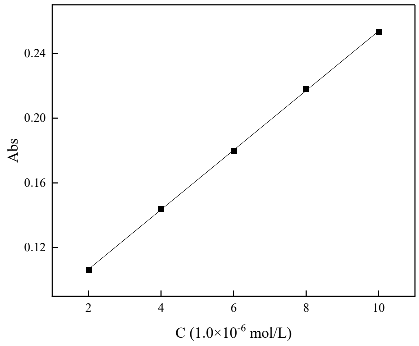

Figure S6. Standard curve of 2,4-dihydroxy-4'-ethoxydibenzophenone

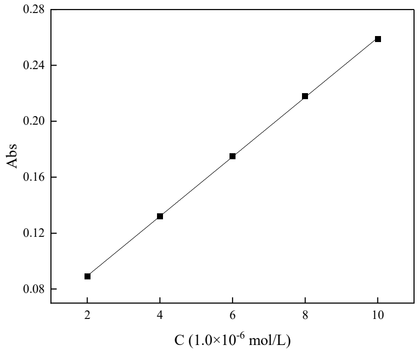

Figure S7. Standard curve of 2,4-dihydroxy-4' -methyldibenzophenone

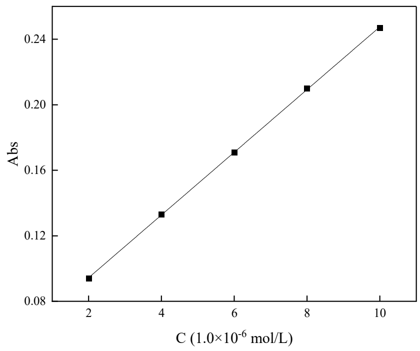

Figure S8. Standard curve of 2,4-dihydroxy-4' -ethyl dibenzophenone

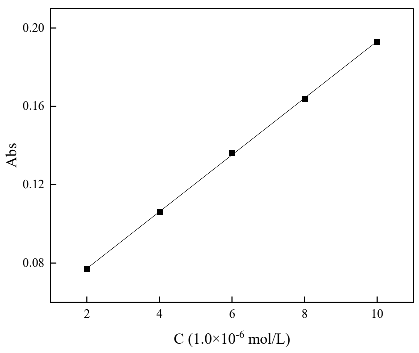

Figure S9. Standard curve of 2,4-dihydroxy-4'-tert-butyldibenzophenone
